# Characterization of light chain c-terminal extension sequence variant in one bispecific antibody

**DOI:** 10.3389/fchem.2022.994472

**Published:** 2022-09-20

**Authors:** Jun Lin, Mengyu Xie, Dan Liu, Zhen Gao, Xiaoyan Zhao, Hongxia Ma, Sheng Ding, Shu mei Li, Song Li, Yanling Liu, Fang Zhou, Hao Hu, Tao Chen, He Chen, Min Xie, Bo Yang, Jun Cheng, Mingjun Ma, Yanyang Nan, Dianwen Ju

**Affiliations:** ^1^ Department of Biological Medicines & Shanghai Engineering Research Center of Immunotherapeutics, Fudan University School of Pharmacy, Shanghai, China; ^2^ Genor Biopharma Co., Ltd., Shanghai, China

**Keywords:** light chain, non-reduced CE-SDS, sequence variant, LC-MS/MS, aberrant splicing

## Abstract

Protein modifications such as post-translational modifications (PTMs) and sequence variants (SVs) occur frequently during protein biosynthesis and have received great attention by biopharma industry and regulatory agencies. In this study, an aberrant peak near light chain (LC) was observed in the non-reduced capillary electrophoresis sodium dodecyl sulfate (nrCE-SDS) electrophoretogram during cell line development of one bispecific antibody (BsAb) product, and the detected mass was about 944 Da higher than LC. The corresponding peak was then enriched by denaturing size-exclusion chromatography (SEC-HPLC) and further characterized by nrCE-SDS and peptide mapping analyses. *De novo* mass spectra/mass spectra (MS/MS) analysis revealed that the aberrant peak was LC related sequence variant, with the truncated C-terminal sequence “SFNR” (“GEC”deleted) linked with downstream SV40 promotor sequence “EAEAASASELFQ”. The unusual sequence was further confirmed by comparing with the direct synthetic peptide “SFNREAEAASASELFQ”. It was demonstrated by mRNA sequencing of the cell pool that the sequence variant was caused by aberrant splicing at the transcription step. The prepared product containing this extension variant maintained well-folded structure and good functional properties though the LC/Heavy chain (HC) inter-chain disulfide was not formed. Several control strategies to mitigate the risk of this LC related sequence variant were also proposed.

## Introduction

Fusion proteins, monoclonal antibodies (mAbs), bispecific antibodies (BsAbs), etc., which are produced by Chinese hamster ovary cell (CHO), SP2/0 or other engineered cells, may contain many kinds of post-translational modifications (PTMs) (Yu et al., 2006; Dick et al., 2008; Raju, 2008; Wypych et al., 2008; Tsubaki et al., 2013; Ecker et al., 2015; Dashivets et al., 2016; Wei et al., 2017) and sequence variants (SVs) (Li et al., 2017; Wong et al., 2018). SVs normally include any unexpected amino acid changes during protein expression. These SVs could be induced by many mechanisms such as deoxyribonucleic acid (DNA) mutations, transcriptional errors and translational errors during the synthesis process of proteins (Wong et al., 2018). For DNA mutations, incorrect base incorporations are observed especially at stressed conditions during replication. Dorai et al. reported a 10% of sequence variant from phenylalanine to leucine in a recombinant protein expressed by CHO-K1 cells with a glutathione synthetase (GS) system (Dorai et al., 2007). It was also reported Tyr376Gln mutation occurred at the DNA level in some sub-clones of stable CHO expression cell lines and the variants decreased along with cell age (Harris et al., 1993). For translational errors, there are multiple mechanisms such as transfer ribonucleic acid (tRNA) misacylation, tRNA mischarging and codon-anticodon mispairing. The occurrences of translational errors are sometimes codons and culturing conditions dependent (McClendon et al., 2006; Yu et al., 2009; Feeney et al., 2013; Lin et al., 2019; Boddapati et al., 2020; Thakur et al., 2021). Thakur et al. found the E262K mutation occurred when the triplet codon “AAG” was used instead of“GAG” (Thakur et al., 2021). Several serine similar amino acids such as aspartic acid, tyrosine, and arginine would bind with seryl-tRNA synthetase (SerRS) and thus induce translation error (McClendon et al., 2006). Misincorporation from valine to isoleucine due to valine starvation during the cultivation was also reported, while feeding valine would mitigate this translation error (Lin et al., 2019).

Different from DNA mutations and translation errors, transcriptional errors such as base substitution, nucleotide skipping/insertion, splicing errors for intron removal and exon skipping occur during RNA transcription and mRNA maturation (Kotia and Raghani, 2010; Yang et al., 2010; Zeck et al., 2012; Zhang et al., 2012; Scott et al., 2014; Huang et al., 2017; Li et al., 2017; Spahr et al., 2018; Wong et al., 2018; Harris et al., 2019; Zhang et al., 2020). These transcription errors would sometimes cause sequence extension (Kotia and Raghani, 2010; Zhang et al., 2012; Scott et al., 2014; Huang et al., 2017; Harris et al., 2019). N-terminal extensions of HC, which were caused by incomplete cleavage of the signal peptides were reported in several mAb products (Kotia and Raghani, 2010; Huang et al., 2017). Reading through stop codons would also make the sequence extended and molecular weight increased (Zhang et al., 2012; Harris et al., 2019). Scott et al. and Spahr et al. found mass extensions by incorporation either part of the light chain vector sequence or the downstream selection cassette promoter to the C-terminal region of the HC (Scott et al., 2014; Spahr et al., 2018). Most of the reported extension occurred at C-terminal of crystallizable fragment (Fc) or HC, and the LC C-terminal extensions were seldom reported. Furthermore, the questions whether these variants would have impact on the structures and functions, and how to detect these trace level variants and set good control strategies have also received great attentions. Mascot Error Tolerant Search (ETS) (Mascot-ETS), automated false positive removal, *de novo* analysis and other strategies were utilized to analyze the trace level sequence variants (Yang et al., 2010; Zeck et al., 2012; Li et al., 2017). To guide biopharmaceutical development, a general SV control limit of 0.1% for each amino acid site was proposed (Zhang et al., 2020). However, since types of SVs are quite different, it sometimes requires case by case analysis before setting rational control strategies.

In this work, we characterized one LC extension sequence variant in the bispecific antibody cell pools. Relying on techniques of nrCE-SDS, denaturing SEC-HPLC, protein mass analysis, peptide mapping-MS/MS and mRNA sequencing, the aberrant peak in nrCE-SDS electrophoregrams (e-grams) was confirmed to be sequence variant linking the truncated C-terminus of LC with downstream SV40 promotor sequence. Though the LC/HC inter-chain disulfide was not formed, the antibody with LC extension had similar thermostability and functional activities compared to its counter-part with normal LC.

## Materials and methods

### Reagents

Formic acid (FA), Dynamis™ medium and CD-CHO medium were purchased from Thermo Fisher Scientific (Pittsburg, PA, United States). Trifluoroacetic acid (TFA) was obtained from TCI (Shanghai, China). Dithiothreitol (DTT) was purchased from Adamas (Shanghai, China). Acetonitrile, iodoacetamide (IAM), urea, β-mercaptoethanol, guanidine hydrochloride, ammonium carbonate, goat anti-human IgG(Fab) and trypsin were purchased from Sigma-Aldrich (St. Louis, MO, United States). Lys-C was obtained from Wako (Osaka, Japan). Peptide:N-glycosidase F (PNGase F) were purchased from New England BioLabs (Ipswich, Massachusetts, United States). IgG Purity and Heterogeneity Kit were purchased from SCIEX (CA, United States). 1% Methyl Cellulose, 0.5%Methyl Cellulose, pI Marker 5.85, pI Marker 9.77 were purchased from Protein Simple (San Francisco, California, United States). Pharmalyte 3–10 was purchased from Cytiva (MA, United States). Epidermal Growth Factor Receptor (EGFR) was from SinoBiological (Beijing, China). The synthesized peptide was prepared by GenScript (Nanjing, China). All the reagents used in our experiments were reagent grade or higher purity.

### Protein expression and purification

The bispecific antibody sequence contained vectors were transfected into CHO-K1 cells and then antibiotic selection was applied for screening stable cell pools. For the BsAbs production, cells were cultivated in Dynamis™ medium for 14 days and harvested by centrifugation. Monoclonal antibodies mAb1 and mAb2 were transiently expressed by CHO-K1 cells and cultivated in CD-CHO production medium for 10 days. For purification, all the proteins were bound and eluted from MabSelect SuRe protein A column (Cytiva, MA, United States), then buffer exchanged into 10 mM sodium acetate solution.

### Non-reduced capillary electrophoresis sodium dodecyl sulfate and reduced capillary electrophoresis sodium dodecyl sulfate analysis

Non-reduced CE-SDS and reduced CE-SDS analysis were carried out using PA800 Plus System (SCIEX, CA, United States). The non-reduced samples were prepared by mixing the protein solution with SDS-MW sample buffer and iodoacetamide, then incubation at 70°C for 10 min. The reduced samples were prepared by mixing the protein solutions with SDS-MW sample buffer and β-mercaptoethanol, then incubation at 70°C for 10 min. The non-reduced samples and reduced samples were both injected at 10 kV for 25 s while separated at 15 kV for 40 min and 30 min, respectively. All the data were acquired by 32 Karat software (SCIEX, CA, United States) and then analysed by Waters Empower 3 Enterprise software (Waters, MA, United States).

### Denaturing SEC-HPLC fraction

Denaturing SEC-HPLC fraction was carried out using an Agilent 1260 HPLC system (Agilent Technologies, Santa Clara, CA) with Waters XBridge BEH 200Å SEC column (Waters, MA, United States). Samples were mixed with 6.4 M Guandine HCl and heated at 37°C for 1 h. The mobile phase was 100 mM sodium phosphate and 100 mM sodium chloride, pH 6.8, and the flow rate was 1.0 ml/min. Fraction-1 was collected from 7.20 min to 7.80 min while fraction-2 was collected from 9.05 min to 9.70 min by monitoring the absorbance at 280 nm. Data was analysed by Agilent ChemStation software (Agilent Technologies, Santa Clara, CA).

### Intact mass analysis by reverse phase chromatography-mass spectra

The intact mass and reduced mass of protein samples were determined using an Waters Synapt Q-TOF (Waters, MA, United States) connected to an Waters H-Class Bio equipped with a Waters BioResolve RP column (Waters, MA, United States) heated to 70°C. Mobile A was 0.05% trifluoroacetic acid (TFA) and mobile B was 0.05% TFA in acetonitrile (ACN). The mobile B gradient increased from 20% to 32% in 1 min and then 32%–75% in 14 min. Capillary voltage and source temperature were set at 3.5 kV and 120°C while scanning range was 500–4000 m/z. The mass spectrometric raw data was deconvoluted by the Waters UNIFI 1.9 software (Waters, MA, United States).

### Peptide mapping analysis by LC-MS/MS

Reduction and alkylation were conducted for protein samples under denaturing conditions by dissolving protein samples with 10 mM dithiothreitol (DTT), 25 mM iodoacetamide (IAM) and denaturing buffer (6 M Gdn-HCl, 50 mM Tris-HCl at pH 7.5). The treated samples were sequentially digested with endoproteinase Lys-C (Lys-C) and trypsin or Lys-C only. For two-step enzyme digestion, samples were first digested at 37°C with an Lys-C: protein ratio of 1:50 (w/w) and the digestion time was 1 h. Trypsin was then added into the samples with an enzyme: protein ratio of 1:20 (w/w) and the digestion time was 2 h. For Lys-C only digestion, samples were digested at 37°C for 2 h with an Lys-C: protein ratio of 1:20 (w/w). The digested samples were analysed using an Waters Acquity UPLC (Waters, MA, United States) coupled with a Thermo Fisher Q Exactive HF-X equipped with an ESI source (Thermo Fisher, Massachusetts, United States). The digested samples were separated with a Waters UPLC CSH130 C18 column Waters, MA, United States) witn the column set at 50°C. Mobile phase A was 0.1% FA in water, and mobile phase B was 0.1% FA in ACN. Peptides were separated using a mobile phase B gradient of 1%–5% in 6 min, 5%–15% in 22 min, 15%–25% in 45 min, 25%–37% in 20 min and 37%–50% in 5 min. Spray voltage and capillary temperature were set at 3.8 kV and 320°C while scanning range was from 200 to 2000 m/z for full MS and normalized collision energy (NCE) 30 was set for MS2 acquisition mode. Raw data was acquired using Thermo Fisher Xcalibur 4.2 software, and database searching was conducted in Thermo Fisher BioPharma Finder 3.0 (Thermo Fisher, Massachusetts, United States).

### SEC-HPLC analysis

SEC-HPLC analysis was carried out using a Waters Alliance e2695 HPLC system (Waters, MA, United States) with Waters XBridge BEH 200Å SEC column (Waters, MA, United States). The mobile phase was 100 mM sodium phosphate and 100 mM sodium chloride, pH 6.8 and the flow rate was 0.5 ml/min. Samples were measured by absorbance at 280 nm. Data was analysed by Waters Empower 3 Enterprise software (Waters, MA, United States)

### Imaged capillary isoelectric focusing analysis

iCIEF analysis was conducted on iCE3 system (ProteinSimple, CA, United States). The samples were prepared by mixing the protein solutions, 1% methyl cellulose, Pharmalyte 3–10, *pI* marker 5.85, 9.77 and water. The mixed samples were pre-focused at 1,500 V for 1 min and then focused at 3,000 V for 8 min with detection wavelength of 280 nm. Data was acquired and analysed by Waters Empower 3 Enterprise software (Waters, MA, United States).

### UNcle analysis

Melting temperature (Tm)/Aggregation temperature (Tagg) analysis was conducted on the UNcle system using mode of differential scanning fluorimetry (DSF) and static light scattering (SLS). Scanning range from 25 to 95 °C with a temperature ramp of 0.5°C/min was applied for the both assays and SLS was measured at 266 nm. Tm and Tagg were analysed and calculated by the UNcle Analysis Software (Unchained Labs, CA, United States).

### Antigen binding activity analysis

96-wells immunoplates were coated with epidermal growth factor receptor (EGFR) and incubated overnight at 4°C. The coated wells were blocked with 1% bovine serum albumin (BSA) in phosphate-buffered saline, 0.1% Tween 20 (PBST) at 25°C for 2 h. The plates were washed and serial dilutions of samples were added to the wells. After incubation at 25°C for 90 min, the plates were washed and then added with goat anti-human IgG antigen-binding fragment (Fab) antibody labelled with horseradish peroxidase (HRP) for detection. The data was acquired by SpectraMax M5 plate reader (Molecular devices, CA, United States) at the wavelength of 450 nm and the four-parameter logistic curves were fitted by Softmax Pro 6.2.2 Software (Molecular devices, CA, United States).

### Neonatal Fc Receptor (FcRn) binding activity analysis

FcRn binding activity analysis was carried out using Biacore T200 system (Cytiva, MA, United States). Human FcRn was immobilized on a CM5 sensor chip with target level of about 100 RU. The running buffer was 20 mM sodium phosphate, pH6.0, 150 mM NaCl, 0.005% polysorbate 20. Samples were 2 fold serially diluted from 1000 nM to 62.5 nM, respectively. Then single cycle kinetics was applied with contact time of 50 s for the ascending 5 concentrations and dissociation time of 100 s. The acquired curves were fitted with steady state affinity model and dissociation value (KD) was calculated.

## Results

### Aberrant near-light chain peak was observed in non-reduced capillary electrophoresis sodium dodecyl sulfate e-gram with its detected mass 944 Da higher than light chain by mass analysis

**FIGURE 1:**
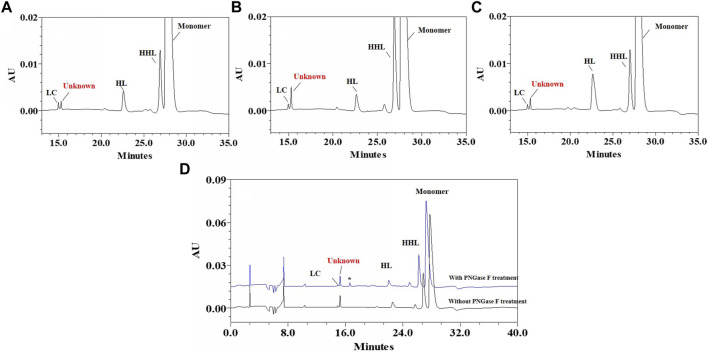
Non-reduced CE-SDS e-grams of BsAb cell pools with unknown peak. **(A)**: Zoomed-in nrCE-SDS e-gram of Cell Pool 1; **(B)**: Zoomed-in nrCE-SDS e-gram of Cell Pool 2; **(C)**: Zoomed-in nrCE-SDS e-gram of Cell Pool 3; **(D)**: nrCE-SDS e-gram of Cell Pool 2 with and without PNGase F treatment; *: PNGase.

**FIGURE 2:**
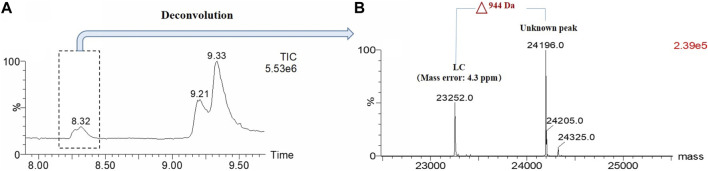
RP-MS based mass analysis of BsAb Cell Pool 2. **(A)**: Total ion chromatogram (TIC) of Cell Pool 2; **(B)**: Deconvoluted mass of LC related peaks. One unknown peak with the mass about 944 Da higher than LC was observed.

When non-reduced CE-SDS (nrCE-SDS) analysis was performed to assess different cell pools of one bispecific antibody (BsAb), an aberrant peak near light chain (LC) was observed, as shown in Figure 1. The migration time of this peak did not change after PNGase F treatment, which indicated the peak was not from the Fc related fragment containing N-glycans. Since the BsAb was constructed by fusing monoclonal antibody (mAb) with nanobody at the N-terminus, our first hypothesis was that this peak may be nanobody related fragments. However, when we compared the electrophoregrams (e-grams) of different cell pools, good correlations of this peak and heavy chain-heavy chain-light chain (HHL) peak were observed. Especially for cell pool 2, the percentages of this unknown peak and HHL peak were both very high, as shown in Figure 1B. The phenomenon indicated that the unknown peak might also be light chain (LC) related but failure to form covalent bond with heavy chain (HC). Then the reverse phase-mass spectrometry (RP-MS) analysis of cell pool 2 sample was performed and LC related peaks were detected among the retention time of 8.2 min–8.4 min, as shown in Figure 2. The corresponding deconvolution mass profile showed two major components, including LC and another LC+944 Da peak. The exact mass assignment excluded either fragmentation from nanobody or HC. The possibility of LC with residual signal peptide or side chain modification were also ruled out. Since the LC+944 Da peak co-migrated with LC in RP-MS, the peak was most likely a LC related variant. Next the unknown peak would be enriched and characterized by applying different kinds of analytical techniques.

### Peak fractioned by denaturing SEC-HPLC and characterized by de novo analysis and comparing with synthetic peptide

**FIGURE 3:**
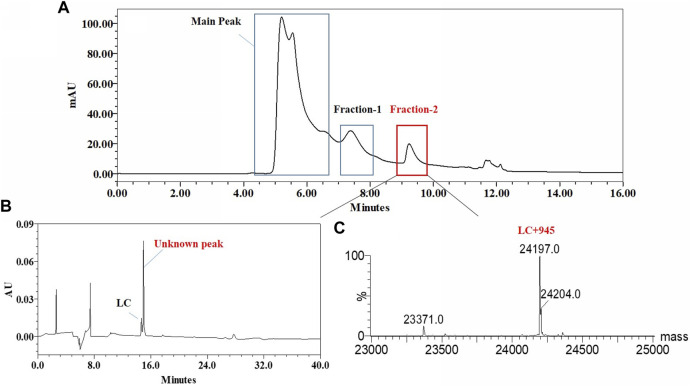
Fraction and identification of LC related Peaks. **(A)**: LC related peaks (Fraction-2) of Cell Pool 2 were fractioned by denaturing SEC-HPLC; **(B)**: nrCE-SDS profile of fractioned peaks, high level of near-LC unknown peak in e-gram was observed; **(C)**: deconvoluted mass of Fraction-2, the detected mass was about 945 Da higher than the theoretical value of LC.

**FIGURE 4:**
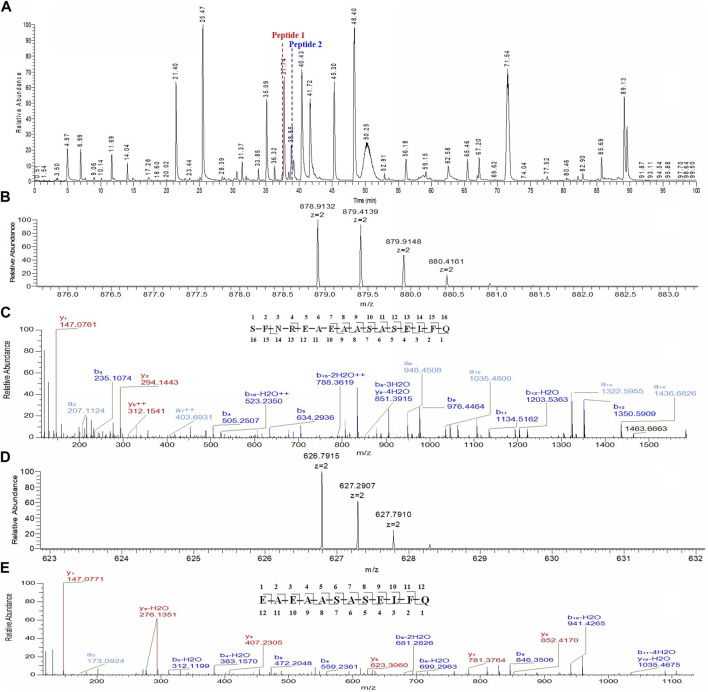
Lys-C/Trypsin peptide mapping and tandem mass analysis of Fraction-2. **(A)**: reduced Lys-C/Trypsin peptide mapping TIC of Fraction-2, most of the peptides were identified except Peptide 1 and Peptide 2; **(B)**: MS profile of Peptide 1; **(C)**: MS/MS profile of Peptide 1, *de novo* sequencing yielded the peptide sequence of “SFNREAEAASASELFQ,” with the observed fragment ions labeled; **(D)**: MS profile of Peptide 2; **(E)**: MS/MS profile of Peptide 2, *de novo* sequencing yielded the peptide sequence of “EAEAASASELFQ,” with the observed fragment ions labeled.

**FIGURE 5:**
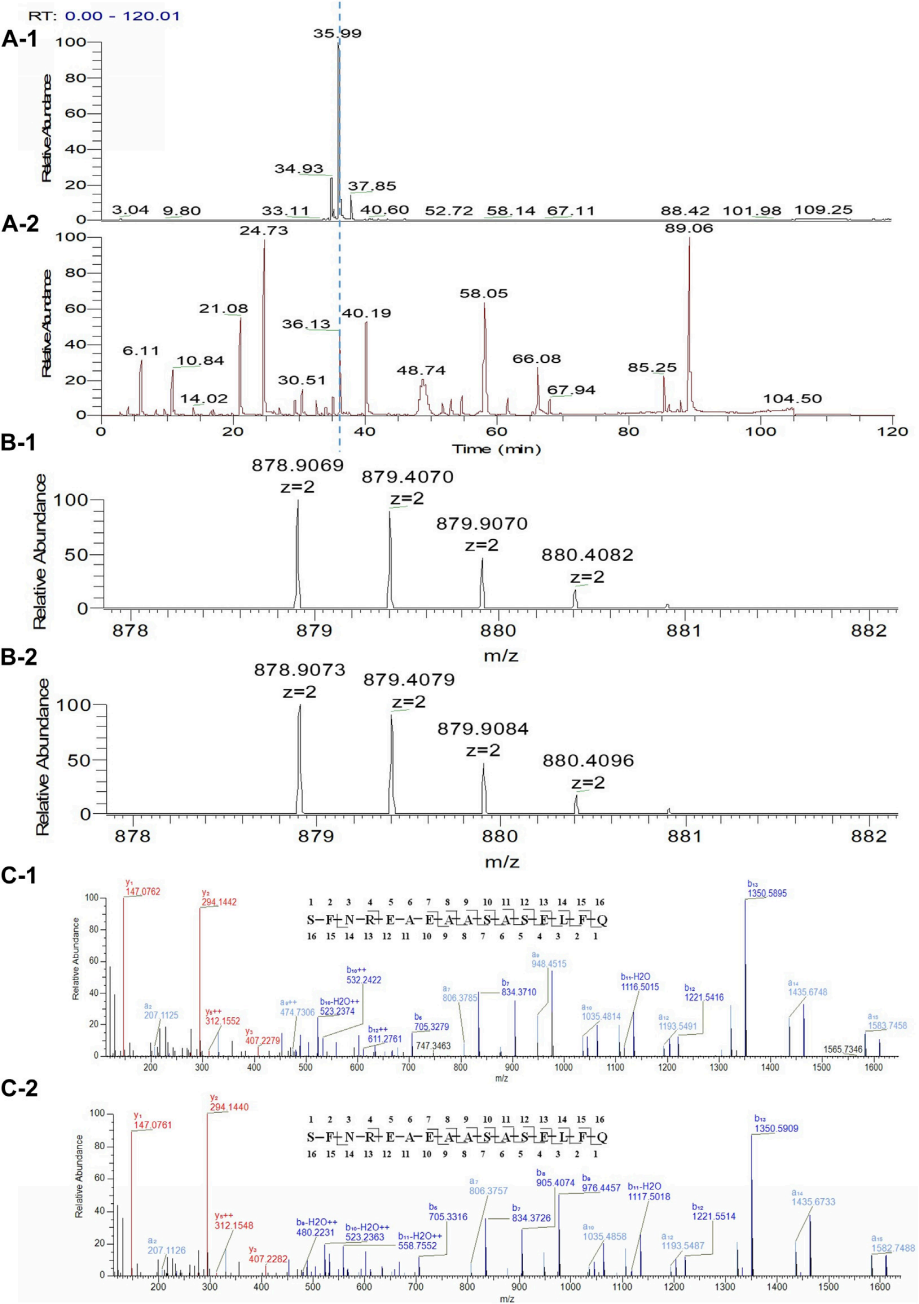
LC C-terminal extension confirm by comparing with synthetic peptide. **(A-1)**: Reduced Lys-C peptide mapping TIC of Fraction-2; **(A-2)**: Reduced Lys-C peptide mapping TIC of the synthetic peptide (SFNREAEAASASELFQ); **(B-1)**: MS profile of the Peptide 1 from Fraction-2; **(B-2)**: MS profile of the synthetic peptide (SFNREAEAASASELFQ); **(C-1)**: MS/MS profile of the Peptide 1 from Fraction-2; **(C-2)**: MS/MS profile of the synthetic peptide (SFNREAEAASASELFQ).

The unknown peak was first found in non-reduced CE-SDS analysis, but it was difficult for this micro-fluid technique to perform direct fraction. A similar size-based denaturing size exclusion chromatography(SEC-HPLC) method was developed after screening the guanidinium hydrochloride (GdnHCl) concentration at 5.3 M, 6 M and 6.4 M. All the conditions showed no differences (Data not shown) and the condition of 6.4 M GdnHCl was applied for the peaks collection. Based on the separation mechanism, 2 peaks later than the main peak were collected, which were named Fraction-1 and Fraction-2. As shown in Figure 3, unknown peak was enriched in Fraction-2 and then confirmed by nrCE-SDS and RP-MS analysis (Data of Fraction-1 not shown). Through nrCE-SDS analysis, high level of the near-LC unknown peak was observed with the percentage higher than 70%. The major mass of Fraction-2 was about 945 Da higher than LC through intact mass analysis. For the LC-MS/MS analysis, the normalized collision energy (NCE) parameters were optimized at NCE 27, NCE 30 and NCE 35 to find the condition with most abundant b/y ion fragments which favoured sequence *de novo* analysis. At the optimized condition of NCE 30, the Lys-C/trypsin digestion coupling with peptide mapping tandem MS analysis was further performed for the Fraction-2 sample. Most of the peptide profiles were in alignment with the sequence of LC except unique Peptide 1 and Peptide 2 which couldn’t be identified. By comparing the MS/MS spectrum of Peptide 1 and Peptide 2, abundant identical b ions and y ions were observed, which indicated there might be partial sequence overlap for Peptide 1 and Peptide 2. Peptide 1 was 504.2434 Da higher than that of Peptide 2, which was exactly the molecular mass of sequence “SFNR”. As shown in Figures 4–C, the mass difference between b4 and b5 is 129.0429 Da, which corresponds to the mass of the residue glutamate. Furthermore, by zooming in the MS/MS spectrum, b6 to b15 ions were also found and corresponding residues were identified to be “EAEAASASELF” considering their mass difference separately. Besides b-ions, there were also y1 ion and y2 ion, according to the mass differences of y-ions, the extra C-terminal sequences were identified as“FQ”. Peptide 1 was thus speculated to be “SFNREAEAASASELFQ” by *de novo* analysis. All the matched fragment ions were labeled in Figure 4. Following the same procedure, Peptide 2 was *de novo* analyzed and the amino acid sequence was speculated to be “EAEAASASELFQ”. All the matched fragment ions were also labeled in Figure 4. According to the *de novo* sequencing results, Peptide 1 and Peptide 2 had the identical sequence of “EAEAASASELFQ”, while the additional “SFNR” of peptide 1 was most likely from the C-terminal sequence “SFNRGEC” of kappa LC. Since the sequence of “SFNREAEAASASELFQ” had one trypsin digestion site between arginine and glutamic acid, so Peptide 2 was most likely to be truncated form of Peptide 1 by trypsin digestion. Furthermore, since the first 4 b ions were relatively few for MS/MS spectrum of Peptide 1, one synthetic peptide with the sequence “SFNREAEAASASELFQ” was prepared to directly confirm the exact sequence. Trypsin specifically cleaves peptide bonds on the carboxyl side of lysine and arginine, while Lys-C only cleaves peptide bonds on the carboxyl side of lysine. By comparison with trypsin, Lys-C digestion generates smaller number but longer peptides. If trypsin digestion was performed for the Fraction-2 sample, 2 different peptides of “SFNR” and “EAEAASASELFQ” would be generated respectively, while for Lys-C digestion, only peptide of “SFNREAEAASASELFQ” would be generated. To keep the integrity of peptide 1, Lys-C digestion instead of Lys-C/Trypsin digestion was performed for the Fraction-2 sample. The digestion sample and the synthetic peptide were compared for MS based peptide mapping analysis. As shown in Figure 5, the synthetic peptide had the same migration time as Peptide 1 from Fraction-2. Furthermore, Peptide 1 showed exactly identical MS and MS/MS patterns as the synthetic peptide. The results demonstrated that the aberrant near-LC peak observed in the nrCE-SDS e-gram was a LC C-terminal extension variant with the ending sequence of “SFNREAEAASASELFQ”. Meanwhile, considering the mutation from original sequence of “SFNRGEC” to “SFNREAEAASASELFQ”, the LC mass would increase by about 945 Da, which was quite in accordance with the inceptive observation of cell pool mass analysis.

### Light chain c-terminal extension due to aberrant splicing was confirmed by mRNA sequencing

**FIGURE 6:**
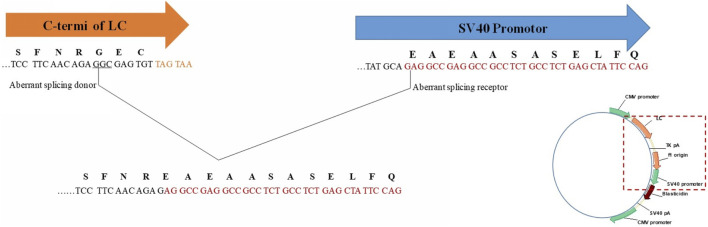
Aberrant splicing induced LC C-terminal extension C-terminus of LC (TCCTTCAACAGAGGCGAGTGT) as aberrant splicing donor and SV-40 promotor (TATGCAGAGGCCGAGGCCGCCTCTGCCTCTGAGCTATTCCAG) as aberrant splicing receptor.

This LC C-terminal extension was first speculated due to read-through of termination codon (Zhang et al., 2012). However, for the extended sequence of “SFNREAEAASASELFQ”, the original LC C-terminal sequence only remained “SFNR”, which meant change occurred before reading to termination codon. Thus, the most likely cause to this change might be aberrant splicing during transcription, especially part of LC C-terminal sequence linked with the downstream vector sequence. By searching the downstream vector sequence, “EAEAASASELFQ” related codons were found in SV-40 promotor, which was commonly used as a transcription element to enhance the expression of plasmids in transfected cells though promotor itself would not express any proteins. By performing mRNA sequencing of Cell Pool 2, low percentage of aberrant splicing was observed. When aligned with plasmid map, it was speculated to be the mRNA alternative splicing of light chain 3′-terminal ligating to a SV40 promoter component. C-terminal of LC (TCC​TTC​AAC​AGA​GGC​GAG​TGT) as aberrant splicing donor and SV-40 promotor (TAT​GCA​GAG​GCC​GAG​GCC​GCC​TCT​GCC​TCT​GAG​CTA​TTC​CAG) as aberrant splicing receptor, the mutant was formed by aberrant splicing and linking the above underlined parts, as shown in Figure 6. Similar phenomenon was reported by Spahr et al. (2018), however, that case was about Fc or heavy chain extension, and only occurred when the codon “GAU” for glycine was utilized. We found the similar aberrant splicing induced extension could also occur at C-terminus of LC. However, splicing site codon optimization by replacing “GAG” for glycine in our case with other codons did not eliminate the phenomenon. Furthermore, different cell pools with the same codon produced different percentages of variants, which indicated the codon might not be the only influencing factor and more studies should be performed to reveal the exact splicing mechanism.

### Structural and functional activities evaluation for light chain c-terminal extension antibody variant

**FIGURE 7:**
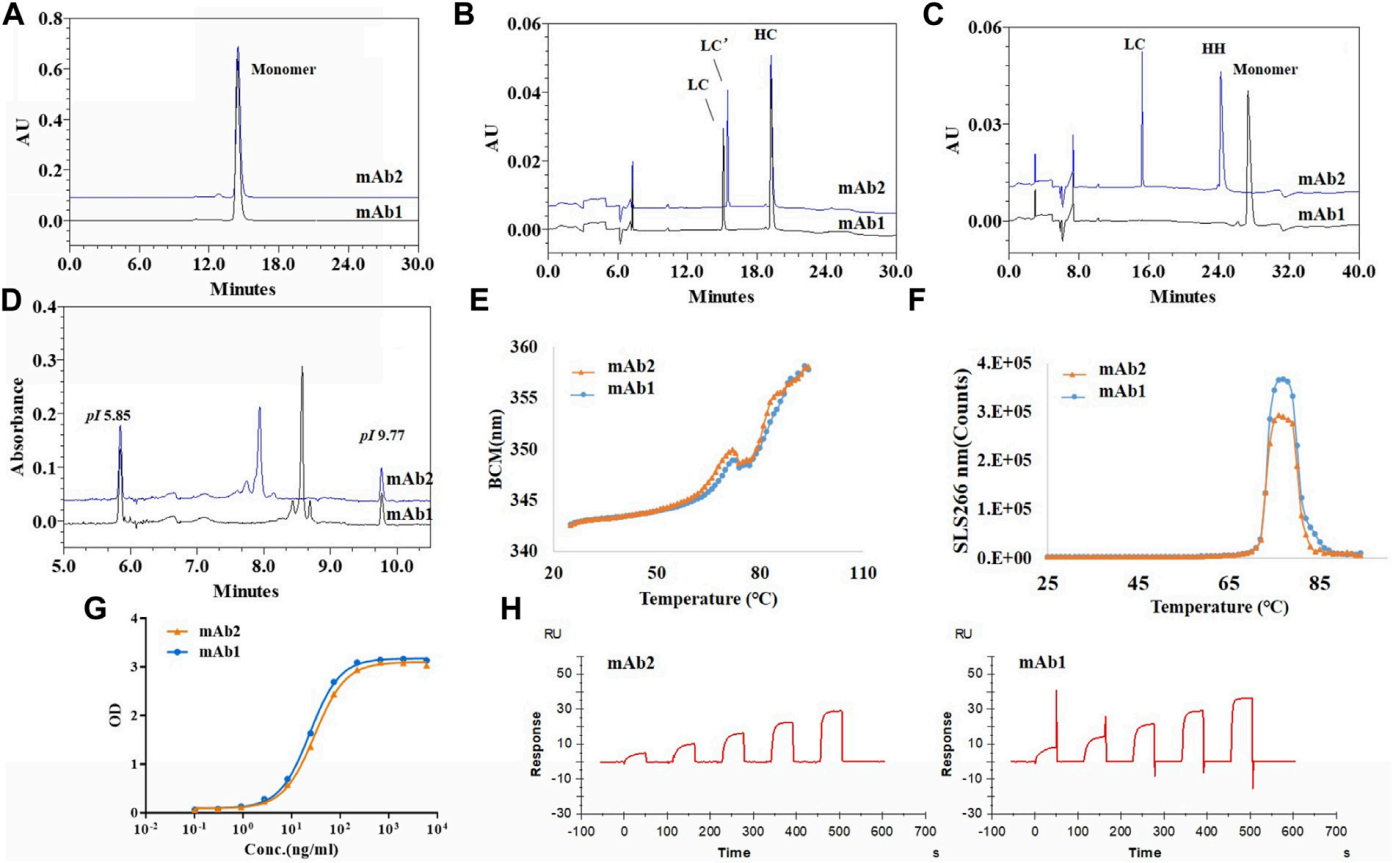
Structural and functional characteristics of antibody with LC C-termial extension (mAb2) and control antibody with normal LC (mAb1). **(A)**: SEC-HPLC profiles of mAb1 and mAb2; **(B)**: rCE-SDS profiles of mAb1 and mAb2; **(C)**: nrCE-SDS profiles of mAb1 and mAb2; **(D)**: pI and charge profiels of mAb1 and mAb2 by iCIEF; **(E)**: Tm analysis of mAb1 and mAb2 by UNcle intrinsic fluorescence scanning; **(F)**: Tagg analysis of mAb1 and mAb2 by UNcle static light scattering; **(G)**: Antigen binding curves of mAb1 and mAb2 by ELISA; **(H)**: FcRn binding activities by SPR. No LC/HC inter-chain disulfide was formed for mAb2, which brought some purities differences but similar functional properties when compared to mAb1. mAb1, control antibody with normal LC; mAb2, Antibody with LC C-terminal extension; LC’: C-terminal extended LC.

To evaluate the impact of LC C-terminal extension on the structural and functional properties of antibody, one antibody with LC C-terminal extension (mAb2, IgG1κ) and one control antibody with normal LC (mAb1, IgG1κ) were prepared and side by side compared. All the results were summarized in Table 1 and the comparing profiles were shown in Figure 7. For the size based purities, mAb2 had slightly higher percentages of aggregates compared to mAb1. mAb2 and mAb1 both had high LC + HC purities (>98%), though the “LC” of two mAbs were different considering their variant migration time. Interestingly, only LC’ and HC-HC instead of monomer was observed in the nrCE-SDS e-gram for mAb2. It could be explained that the C-terminal LC extension variant did not contain C214 (Eu numbering), so no disulfide bond between HC and LC was formed for mAb2. For *pI* and charge purity, mAb2 had lower *pI* and higher acidic peaks compared to mAb1, which indicated the charge differences of the two and favoured separating the variant through ion exchange chromatography in the purification step. Though purities differences observed, mAb1 and mAb2 had very similar thermostability and functional properties. The melting temperature (Tm) and aggregation temperature (Tagg), antigen binding activity and neonatal Fc receptor (FcRn) binding activity were exactly same for the 2 mAbs. So it was supposed that though the inter-chain disulfide was not formed for mAb2, heavy chain could also interact with light chain through ionic bonds (salt bridges), hydrogen bonds, and hydrophobic interactions (Orcutt et al., 2010; Brinkmann and Kontermann, 2017) to help mAb2 maintain well-folded structure and favourable functions at the native condition.

**TABLE 1:** Properties comparison of normal antibody and LC C-terminal extension variant.

Properties	mAb1 (control antibody with normal LC)	mAb2 (antibody with LC C-terminal extension)
Schematic diagram	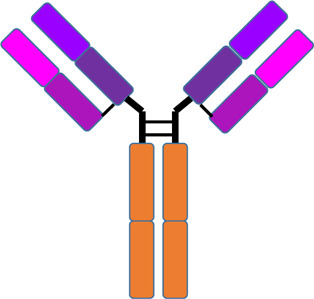	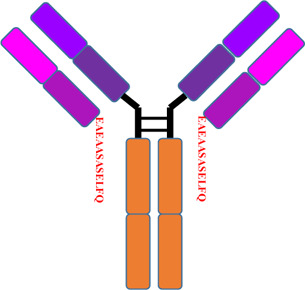
SEC-HPLC Purity (HMWs/Monomer/LMWs)	3.7%/95.5%/0.8%	6.3%/92.8%/0.9%
nrCE-SDS Purity (Monomer)	96.4%	No monomer
rCE-SDS Purity (LC + HC)	98.3%	98.2%
iCIEF Purity (Acidic/Main/Basic)	18.3%/71.4%/10.3%	37.7%/56.6%/5.7%
pI (Theo./Detec.)	8.2/8.6	7.9/7.9
UNcle (Tonset/Tm1/Tm2)	62.3°C/69.2°C/83.8°C	61.0°C/67.4°C/81.0°C
UNcle (Tagg266)	71.1°C	70.8°C
Antigen Binding Activity (EC50)	23.65 ng/ml	29.75 ng/ml
FcRn Binding Activity (KD)	3.13E-07 M	3.19E-07 M

## Discussion

### Retrospective analysis of approved antibody drugs

Since the LC C-terminal extension was far from Complementary Determining Regions (CDRs) and Fc Receptor binding regions, this sequence variant has no influence on the functional properties. However, the impact of this LC related sequence variant on immunogenicity and pharmacokinetics (PK) are still controversy considering it is not natural-structure protein. Whether the same variant exist in approved antibody drugs and what’s the contents are also worthy studying. 16 approved antibody drugs with kappa chain such as Herceptin™, Remicade™ etc. Were investigated by detecting the near-LC aberrant peak through nrCE-SDS analysis and unique peptide of “SFNREAEAASASELFQ” through Lys-C peptide mapping analysis, however, no same LC related variant was found for all these drugs (data not shown). Though we did not extend to more other approved drugs due to limited accessibility, there was high probability that this LC extension sequence variant was first found and reported. So there was no PK, immunogenicity, safety related data could be referred directly. Considering there are some LC C terminal extension bispecific antibodies such as IgG-scFv (LC) on development^31,32^, more and more nonclinical and clinical data would be available in the future for the references, though the sequences were not exactly same.

### Control strategies to mitigate the risk of the variant for development

The quality attribute criticality assessment was performed based on the impact on the potency/efficacy, PK/pharmacodynamics (PD), immunogenicity and safety (CMC Biotech Working Group, 2009). Considering the structure of LC C-terminal extension and our study results, the impact of this LC sequence variant on the potency/efficacy would be low. Non-natural proteins with aberrant structure might induce anti-drug antibody and thus the impact on immunogenicity was given a high score. On the other hand, since this variant was new found, the uncertainty score was given the highest value of 7, thus by multiplying the Impact value with Uncertainty value, the Criticality score could be as high as 112. So the LC related sequence variant would be evaluated as potential critical quality attribute (pCQA). Since the LC related sequence variant was pCQA, several control strategies were performed to lower the risk of this quality attribute. Through different clones screening and cultivation conditions optimization, this LC sequence variant could be reduced to <0.1%. On the other hand, due to charge differences between this sequence variant and its counter-part antibody with normal LC, ion exchange chromatography based purification could favor completely clearance of this variant. Furthermore, nrCE-SDS as the product release method, has good capability to detect the aberrant LC-near peak and control the content of the LC related sequence variant. Peptide mapping tandem MS analysis could identify the unique peptide of “SFNREAEAASASELFQ” and periodically monitor the percentage of the variant. All these combined control strategies would mitigate the risk of the variant in the future drugs developments. These findings thus would help the biopharma industry to realize this kind of LC extension sequence variant and establish more reasonable process and analytics related control strategies.

## Data Availability

The raw data supporting the conclusions of this article will be made available by the authors, without undue reservation.
